# Supplementary material for: Space-time covid-19 Bayesian SIR modeling in South Carolina
Source: PLoS One. 2021 Mar 17;16(3):e0242777. doi: 10.1371/journal.pone.0242777 (PMC7968659; doi:10.1371/journal.pone.0242777)
Supplement: S1 Appendix — (DOCX) [file pone.0242777.s001.docx]

**S1 Appendix**

Nimble code for the model 4

-----------------------------------------------------------------------------------

COVModel4<-nimbleCode({

for (i in 1:M){

remc[i,1]<-0

remD[i,1]<-0

susc[i,1]<-susint[i]

muc[i,1]<-0.001*susc[i,1]

sym[i,1]~dpois(muc[i,1])

asym[i,1]<-0.33*sym[i,1]

LdevC[i,1]<--2*(sym[i,1]*log(muc[i,1]+0.001)-(muc[i,1]+0.001)-lfactorial(sym[i,1]))

}

for (i in 1:M){

for (j in 2: T){

remc[i,j]<-betaRc*sym[i,j]

remD[i,j]<-deaths[i,j]

susc[i,j]<-susc[i,j-1]-sym[i,j-1]-asym[i,j-1]-remc[i,j-1]-remD[i,j-1]

sym[i,j]~dpois(muc[i,j])

asym[i,j]<-0.33*sym[i,j]

log(muc[i,j])<-bet0[j]+log(susc[i,j]+0.001)+bet1*(log(sym[i,j-1]+0.001)+log(asym[i,j-1]+0.001))+bet2*percP[i]+b1[i]

LdevC[i,j]<--2*(sym[i,j]*log(muc[i,j]+0.001)-(muc[i,j]+0.001)-lfactorial(sym[i,j]))

}

muct1[i]<-muc[i,1]

muct2[i]<-muc[i,2]

muct3[i]<-muc[i,3]

muct4[i]<-muc[i,4]

muct5[i]<-muc[i,5]

muct6[i]<-muc[i,6]

muct7[i]<-muc[i,7]

muct8[i]<-muc[i,8]

muct9[i]<-muc[i,9]

muct10[i]<-muc[i,10]

muct40[i]<-muc[i,40]

muct46[i]<-muc[i,46]

muct50[i]<-muc[i,50]

muct60[i]<-muc[i,60]

muct70[i]<-muc[i,70]

remt1[i]<-remc[i,1]+remD[i,1]

remt2[i]<-remc[i,2]+remD[i,2]

remt3[i]<-remc[i,3]+remD[i,3]

remt4[i]<-remc[i,4]+remD[i,4]

remt5[i]<-remc[i,5]+remD[i,5]

remt6[i]<-remc[i,6]+remD[i,6]

remt7[i]<-remc[i,7]+remD[i,7]

remt8[i]<-remc[i,8]+remD[i,8]

remt9[i]<-remc[i,9]+remD[i,9]

remt10[i]<-remc[i,10]+remD[i,10]}

for (j in 1:T){

mucrich[j]<-muc[40,j]

mucchar[j]<-muc[10,j]

muchor[j]<-muc[26,j]

mucbea[j]<-muc[7,j]

mucker[j]<-muc[28,j]

mucand[j]<-muc[4,j]

mucsum[j]<-muc[43,j]

mucflor[j]<-muc[21,j]

}

b1[1:46] ~ dcar_normal(adj[1:L], wei[1:L], num[1:M], tau.b1,zero_mean=1)

for(k in 1:L) {wei[k] <- 1 }

DevC<-sum(LdevC[1:M,1:T])

for (k in 1:T){

bet0[k]~dnorm(0,tau0)}

tau0~dgamma(2,0.5)

bet1~dnorm(0,tau1)

tau1~dgamma(2,0.5)

tau.b1~dgamma(0.01,0.01)

betaRc<-0.1

bet2~dnorm(0,tau2)

tau2~dgamma(2,0.5)

#R0<-exp(bet0)/betaRc

}
